# Research on optimization of personalized recommendation method based on RFMQ model— taking outdoor sports products in cross-border e-commerce as an example

**DOI:** 10.3389/fdata.2025.1680669

**Published:** 2025-10-14

**Authors:** Qianlan Chen, Chupeng Chen, Zubai Jiang, Chaoling Li, Yangxizi Tan, Niannian Li, Bolin Zhou, Bingxian Yang

**Affiliations:** ^1^College of Physical Education and Health, Guangxi Normal University, Guilin, China; ^2^Key Laboratory of Digital Empowerment Economic Development, Guangxi Normal University, Guilin, China; ^3^School of Management Science and Engineering, Guizhou University of Finance and Economics, Guiyang, China; ^4^College of Management, Guilin University of Aerospace Technology, Guilin, China; ^5^School of Economics and Management, Guangxi Normal University, Guilin, China

**Keywords:** cross-border e-commerce, collaborative filtering, segmentation by index, k-means clustering algorithm, RFMQ model, customer segmentation

## Abstract

With the rapid development of the global digital economy, cross-border e-commerce has rapidly emerged and developed at a high speed, and has become a crucial bridge connecting global markets. This research focuses on the cross-border e-commerce sector of outdoor sports products, in response to the common problems in the cross-border e-commerce field, such as “information overload” and “insufficient recommendation accuracy,” a personalized recommendation optimization framework integrating customer value segmentation and collaborative filtering is proposed. Based on the classic RFM model, the purchase quantity indicator (Quantity) is introduced to construct the RFMQ model, thereby more comprehensively characterizing user behavior characteristics. Further, the customer value stratification is achieved by using the indicator segmentation method and the K-means clustering algorithm, and a differentiated collaborative filtering recommendation mechanism is designed based on the segmented groups. Through a five-fold cross-validation experiment, it is shown that the proposed method significantly outperforms the traditional collaborative filtering model in the TOPN recommendation task. Specifically, when the number of recommended products is between 3 and 7, the RFMQ recommendation model based on indicator segmentation performs best in terms of F1 score (for example, when TOPN = 5, the F1 value increases from 0.1709 to 0.3093), and the method based on K-means clustering also shows a stable improvement (with the F1 value reaching 0.267 at the same time). The results indicate that the indicator segmentation method has a significant advantage in smaller recommendation quantity scenarios. This study verifies the effectiveness of the RFMQ model in customer segmentation and recommendation performance optimization, providing an operational solution for e-commerce platforms to implement precise marketing, enhance user stickiness and commercial competitiveness, and is particularly suitable for low-cost and high-efficiency personalized recommendation scenarios of small and medium-sized enterprises.

## 1 Introduction

Cross-border e-commerce, as an emerging foreign trade model, has become an important part of global trade due to its high openness and wide applicability. During the 2019 coronavirus pandemic, the volume of cross-border e-commerce orders increased by 25% (Han and Lai, 2025) from 2020 to 2022. Currently, cross-border e-commerce accounts for approximately 30% of the e-commerce market, with 60% of it being intercontinental transactions (Beretzky et al., 2022). This industry continues to show a vigorous development trend. According to CBEC's prediction, the transaction volume will reach 25 to 30 billion to 300 billion transactions by 2030 (Beretzky et al., 2022).

At the same time, under the influence of multiple factors such as policy support, the promotion of Olympic events, and the dissemination of fitness culture, the number of global sports participants has rapidly increased, driving the continuous growth of the demand for sports equipment (China Insights Consultancy, 2023). China, as a major manufacturing country of sports goods, has occupied 65% of the global production share. Although the Chinese sports equipment market started relatively late (beginning in the late 1980s), it has developed rapidly and has become a pillar of the Chinese sports goods industry in just over 30 years (Wang and Yang, 2015). In the cross-border e-commerce field of outdoor sports products, relying on the global logistics network, characteristic products such as camping equipment, fitness equipment, and outdoor clothing can be efficiently reached by global consumers. However, this field has a wide range of product categories and significant differences in user needs, as well as significant seasonal and situational factors, which place higher requirements on the precision selection of products and personalized recommendations by e-commerce platforms.

Currently, e-commerce platforms generally use search engines to help consumers quickly locate products. Although this method is intuitive, it still has obvious limitations: traditional search engines are mostly based on the RFM model, and their effectiveness highly depends on the accurate expression of users' needs. Once users cannot clearly describe their needs, the search results are likely to deviate from the actual needs, resulting in low information matching efficiency. Moreover, general search services are difficult to cope with the increasingly diverse and personalized needs of consumers (Zhou and Yang, 2020). The “one-size-fits-all” service model has become difficult to adapt to the complex and changing personalized consumption environment.

In this context, personalized recommendation technology provides a more intelligent solution. This technology achieves precise product recommendations by analyzing users' historical behaviors and interest preferences (Hong et al., 2016), which can effectively reduce user choice fatigue, accelerate purchase decisions, and improve transaction efficiency. For cross-border platforms, optimizing recommendation technology not only helps improve user experience but also significantly increases sales and profits. This paper constructs an integrated recommendation system that combines RFMQ customer value assessment, entropy weight method weighting, customer segmentation, and collaborative filtering, further optimizing customer classification and recommendation accuracy, thereby enhancing the platform's competitiveness.

## 2 Literature review

This study is based on the RFM model and the collaborative filtering recommendation algorithm. The RFM model (Recency, Frequency, Monetary) is a classic tool for evaluating customer value, which was proposed by Hughes in the early 1990s (Hughes, 1994) and has been widely applied in marketing and customer relationship management (CRM) fields. This model, by analyzing the customer's recent purchase time, frequency, and spending amount, can effectively assist enterprises in customer segmentation and marketing strategy optimization. However, as the market environment and customer behaviors continue to change, the traditional RFM model gradually shows limitations in terms of dynamic adaptability and stability.

To overcome these limitations, scholars have proposed various improvement schemes. For instance, Zong and Xing (2021) combined the RFM model with the CTS index (service cost) to construct the RFMC model, which enables a more comprehensive assessment of customer value. Chavhan et al. (2025) introduced the delivery rate (D) of customer shopping cart behavior and proposed the R + FMD model to enhance the accuracy of customer segmentation and thereby improve customer retention (Chavhan et al., 2025). Niu and Hao (2019) applied the RFMQ model to alleviate the interference of product category dimensions and improved the efficacy of the recommendation system. These studies show that by introducing diversified indicators, the RFM model can be optimized and better adapted to modern business needs. It is worth noting that although the Monetary (M) indicator represents the total customer consumption amount, the Quantity (Q), which refers to the purchase quantity, still has independent significance. The Q indicator can reflect the intensity of customer purchase frequency, category preference, and fulfillment behavior patterns, and when combined with M, it can more precisely depict customer value. For example, differentiating between “low-frequency high amount” and “high-frequency low amount” and other behavior types, especially in cross-border e-commerce, Q helps identify bulk purchasing, repeat purchase behaviors, or users with specific category preferences, thereby reducing the deviation caused by relying solely on amount and enhancing the granularity of segmentation and the targetedness of recommendations.

Furthermore, Wu et al. (2021) combined the RFM model with the K-Means algorithm to segment the user groups of the T-app community e-commerce platform. They determined the optimal K value by using the concept of the silhouette coefficient and clustered the weighted indicators using the K-Means clustering algorithm. Finally, they classified the customers into different value customer groups (Wu et al., 2021). Zaghloul et al. (2025) further integrated K-Means, RFM, and deep learning models (LSTM, GRU), proposing a new customer retention analysis framework, providing actionable insights for customer retention strategies in e-commerce (Zaghloul et al., 2025). These works indicate that introducing the K-Means clustering method can significantly enhance the analytical capabilities of the RFM model. Applying the K-Means clustering algorithm can significantly improve the accuracy of the RFM model analysis results. Based on this, based on the above progress, this study introduces the Quantity (Q) indicator to construct the RFMQ model and adopts the K-Means clustering and indicator segmentation methods to improve the accuracy and practicality of the traditional RFM model in customer segmentation, especially for the mining needs of multi-dimensional user behavior data in e-commerce platforms.

With the rapid development of the Internet and digital technology, recommendation systems have become an indispensable part of various platforms. From e-commerce, video to news and other application fields (Raza and Ding, 2022), the role of recommendation systems is increasingly prominent, providing users with personalized content and product recommendations, and helping enterprises more accurately capture users' needs and interests (Huang et al., 2022). Customer segmentation as the basis of precise marketing provides a key input dimension for personalized recommendation systems. Especially the multi-dimensional customer value stratification obtained by the RFMQ model can provide more detailed and interpretable user profiles for recommendation systems. For example, customers with high purchase quantity (Q) may tend to purchase in bulk or for specific categories, while customers with high spending amount (M) and recent activity (R) are more likely to be interested in high-end or new product items. These segmentation features significantly enhance the ability to represent users, providing richer signals for subsequent recommendation algorithms.

Existing recommendation systems can be classified into three categories: content-based, collaborative filtering, and hybrid recommendation. Among them, the collaborative filtering recommendation method occupies a dominant position among many recommendation mechanisms, and with the advancement of technology, how to ensure user privacy is protected during the recommendation process has also gradually attracted the attention of researchers. Collaborative filtering recommendation systems are an important method in recommendation algorithms, predicting and recommending items that users may like by collecting and analyzing users' historical behavior data. This method, with its simple theory and practical effectiveness, has been widely applied in many recommendation systems. At the same time, with the development of big data and machine learning technologies, how to better utilize users' attributes and preferences to improve the accuracy and diversity of recommendations has also become a research hotspot.

Collaborative filtering recommendation system, as the core technology of personalized recommendation, has continuously attracted extensive attention from the academic community. Its research direction shows a diversified and in-depth development trend. The user-based collaborative filtering was initially proposed by Goldberg et al. (1992) in the Tapestry system, which is particularly suitable for situations where users have similar behavior patterns. The item-based collaborative filtering focuses on calculating the similarity between items and recommends similar items based on the user's previously liked items. Fan et al. (2014) introduced the KNN algorithm into collaborative filtering to improve the accuracy of recommendations and fill in the missing rating data, achieving the goal of optimization. Shinde and Kulkarni (2011) optimized the calculation process by introducing the K-means clustering algorithm, improving the real-time performance of recommendations and addressing the problem of poor scalability. Chen (2024) combined the K-means clustering algorithm and the collaborative filtering recommendation algorithm, analyzed the characteristics of music learners, thereby obtaining their similarity, to build a music learning resource recommendation model, and classified music learners to quickly provide different learning resource recommendations for different music learners, thereby enhancing students' enthusiasm for autonomous learning. Terui et al. (2025) proposed a collaborative filtering method based on non-negative/binary matrix decomposition, effectively extracting the potential features of users and items through matrix decomposition technology, significantly optimizing the recommendation performance. Müllner et al. (2023) systematically reviewed the differential privacy protection techniques in collaborative filtering, discussing how to effectively protect user privacy information while ensuring the recommendation effect, providing important support for the practical application of this direction. In addition, attribute-aware collaborative filtering models have also received in-depth research. This type of model not only relies on users' historical ratings but also integrates user, item, and rating-related attributes (such as age, price, time, etc.) through mathematical modeling and experimental verification, significantly improving the model's expression ability and scene adaptability (Chen et al., 2020). The above studies have respectively promoted the development of collaborative filtering systems from multiple different perspectives such as matrix decomposition, privacy protection, and multi-attribute fusion, demonstrating the rich technical content and application potential of this field.

In the application level, the e-commerce scenarios represented by online grocery shopping have put forward more complex requirements for recommendation systems, not only needing to meet personalization but also considering the diversity of recommendations and the complementarity between items. For example, when users purchase vegetables, they may also need to be recommended complementary seasonings or ingredients. Traditional collaborative filtering methods often rely too much on historical behaviors, which can easily lead to repetitive recommendations and lack of novelty. Therefore, the attribute-aware CF model that integrates user attributes, behavioral context, and item correlations shows its advantages. It can more comprehensively understand user intentions and the relationships between items, thereby supporting complementary and diverse recommendations.

This study, based on the existing collaborative filtering and customer segmentation research, proposes a customer value segmentation and recommendation method based on the RFMQ model and K-means clustering algorithm. By extending the traditional RFM model with the Quantity (Q) indicator, it effectively identifies user batch purchasing, repeat purchase preferences and other behavioral characteristics, and combines cluster analysis to achieve more refined customer grouping (Ma et al., 2023). Experimental results show that the proposed method significantly outperforms the traditional collaborative filtering model in the Top-N recommendation task, especially showing good applicability in the cross-border e-commerce scenario. This study provides a practical customer segmentation and recommendation framework for e-commerce platforms, improving recommendation accuracy while providing theoretical basis and practical references for multi-dimensional user behavior mining and personalized marketing strategy formulation. Future research directions include introducing dynamic update mechanisms, integrating multi-source heterogeneous data, and exploring the application potential in cross-domain recommendation scenarios.

## 3 Methodology and background

This study's recommendation methodology does not directly apply collaborative filtering algorithms to all customers. Instead, it utilizes customer segmentation results to provide tailored recommendations for different value-oriented groups, while conducting comparative experiments with traditional unsegmented recommendation models. Through experimental validation, we explore whether the combination of “customer segmentation + collaborative filtering recommendation” can effectively enhance recommendation performance, investigating whether the “category-driven recommendation” design approach demonstrates significant improvement in recommendation effectiveness.

In this experiment, the effects of traditional collaborative filtering recommendation, index segmentation based collaborative filtering recommendation and K-means method based collaborative filtering recommendation are compared respectively, so as to select the optimal recommendation algorithm.

### 3.1 Data background

You may insert up to five heading levels into your manuscript as can be seen in “Styles” tab of this template. These formatting styles are meant as a guide, as long as the heading levels are clear, Frontiers style will be applied during typesetting.

#### 3.1.1 Basic data information

Due to the confidentiality of the data by the platform and the privacy protection of the sellers, it is difficult to obtain the transaction data, and it is also hard to find relevant data in the publicly available datasets. Therefore, this article selects the personal data set published on Kaggle. The data collected includes the historical transaction order data of a cross-border e-commerce platform company that mainly deals in outdoor sports products in 2020, serving as the experimental data source for this study. The data reflects the diverse purchasing behavior of cross-border groups. The data consists of 8 variables: Order Number (InvoiceNo), Product Code (StockCode), Product Description (Description), Quantity, Order Date (InvoiceDate), Unit Price (UnitPrice), Customer ID (CustomerID), and Country. The original data contains 541,910 records.

#### 3.1.2 Data preprocessing

During the collection and storage of raw data, noise contamination often occurs. Such interference may compromise data accuracy, integrity, and consistency, creating additional challenges for subsequent processing. To ensure reliable and precise results in customer segmentation analysis, it is essential to clean sample data before implementation by removing noise, missing values, and other potential issues. Below are the specific cleaning procedures applied to this dataset:

(1) Check and handle missing values. Through checking the missing values, there are 1,454 missing values in the Description column and 135,080 missing values in the Customer ID column, while there are no missing values in other columns. Delete the rows containing missing values in these columns.
(2) Processing of duplicate records, finding and deleting duplicate records, the remaining data volume is 401,604.
(3) Handle redundant information, ensure that the Product Code is a 5-digit integer, and process the remaining data volume to 358,277 records.
(4) Redundant information processing to ensure that the product number is a 5-digit integer and the remaining data volume is 358,277.
(5) Invalid order processing, that is, orders with negative quantity, and the remaining data volume is 358,277.
(6) Data selection and conversion: The baseline time point is determined by selecting the latest transaction date from all records in the dataset. For each customer, the interval between their last transaction date and this baseline time point is calculated to determine the Recency metric. Purchase Frequency (Frequency) is measured by counting unique orders per customer, where multiple purchases under the same order number are counted as a single transaction. Monetary Amount (Monetary) is calculated by multiplying the quantity purchased by the unit price for each transaction, then summing these amounts across all transactions using customer IDs. Finally, Quantity (Quantity) is obtained by aggregating the total number of items purchased across all transactions.
(7) Data standardization processing: During the data processing process, this article selects four key indicators that meet the model requirements from the original 8 variables and performs transformation processing. Since these indicators have different units and dimensions, they cannot be directly analyzed, so data standardization processing is required. This article adopts the Z-score standardization method to convert each indicator into data without units. Regarding the negative indicator (Recency), an inverse operation method was adopted to ensure that this indicator has the same direction as the other indicators. After data cleaning and standardization processing, a total of 358,277 valid transaction records and 4,314 valid customers were obtained, laying the foundation for the subsequent customer segmentation analysis. The pre-processing results are shown in Table 1 (due to the large dataset, only some results are presented for illustration).

**Table 1:** Results of RFMQ data preprocessing.

**CustomerID**	**Recency**	**Frequency**	**Monetary**	**Quantity**
13951	−0.82813	−0.1624	−0.14359	−0.13733
13952	−1.24643	−0.29513	0.109919	0.017275
13953	0.855009	0.368544	0.178298	0.329623
13954	0.904806	−0.29513	−0.17247	−0.20206
13955	−0.60902	−0.29513	−0.18396	−0.20038
13956	0.874927	0.103074	−0.10511	−0.13335
13959	0.137931	−0.1624	−0.1614	−0.17231
13960	0.715577	−0.29513	−0.19262	−0.20038
13962	0.705617	−0.29513	−0.19982	−0.21191
13963	−2.14277	−0.02966	−0.15584	−0.15849

A lower Recency score indicates that customers have recently made purchases, reflecting higher engagement levels. A high Frequency score suggests frequent purchasing activity, while Monetary represents total spending on the platform. Customers with high scores are typically considered high-value users. Quantity measures the number of products purchased. These standardized metrics provide clear quantitative parameters for customer segmentation, helping us distinguish different consumer groups and laying the foundation for targeted marketing and personalized recommendations.

### 3.2 Customer segmentation based on the RFMQ model

In the customer value assessment, based on the values of the four indicators of the RFMQ model, customers can be classified into different value levels. To effectively conduct customer segmentation, this paper uses both the K-means clustering customer segmentation method and the indicator segmentation method.

(1) K-means clustering segmentation method

The K-means clustering segmentation method automatically divides customers into multiple groups based on their characteristics (such as purchase frequency, spending amount, purchase quantity, etc.), and can perform group division based on the inherent similarities of the data itself. The core point of this method is to determine the number of clusters K. The commonly used methods for determining the number of clusters include the elbow method and the silhouette coefficient method. The specific introduction is as follows:

①Elbow method

When using the elbow method to determine the number of clusters, the main criterion is the size of SSE (Sum of Squared Errors), and the specific calculation formula is as shown in Equation 1:


(1) $$\begin{array}{l} SSE=\sum_{i=1}^{k}\sum_{p\in C_{i}}|p-m_{i}{|}^{2} \end{array}$$

Among them, *C*_*i*_ represents the nth cluster, *p* represents a certain sample point within the cluster, and *m*_*i*_ represents the centroid of the cluster (the average value of all samples).

The basic idea of the elbow method is as follows: As the number of clusters k increases, the sample distribution becomes more uniform, and at this point, the SSE will gradually decrease. When the number of clusters k is less than the actual number of clusters, the decrease in SSE is significant because as k increases, the degree of aggregation of each cluster increases sharply. However, when k reaches the actual number of clusters, the change in the degree of aggregation obtained by increasing k will become smaller, and the decrease in SSE will also decrease. Then, as k continues to increase, it will stabilize. That is to say, the relationship graph between SSE and k is in the shape of an elbow, and the k value corresponding to this elbow is the true number of clusters for the data.

② Outline coefficient method

For a given sample point, the calculation formula is as follows Equation 2:


(2) $$\begin{array}{l} S\;=\frac{b-a}{\max\left(a,b\right)} \end{array}$$

In Equation 2, a and b, respectively represent the degree of aggregation and the degree of separation. The degree of aggregation indicates the average distance between sample points of *X*_*i*_ within the same cluster, while the degree of separation indicates the average distance between *X*_*i*_ and all sample points in its nearest cluster. The specific calculation method for the nearest cluster is as shown in Equation 3:


(3) $$C_{j}=\arg\frac{1}{n}\sum_{p\in c_{k}}|p-X_{i}{|}^{2}$$

Among them, *p* represents the samples within the given cluster *c*_*k*_. In fact, the cluster that is closest to *X*_*i*_ is selected based on the average distance of all samples in *X*_*i*_ to the given cluster. This point is then used to estimate the distance between this point and the given cluster.

The average silhouette coefficient is obtained by averaging the silhouette coefficients of all the samples. The range of the average silhouette coefficient is [−1, 1], and the higher the average silhouette coefficient, the better the clustering effect. Therefore, the K value with the highest average silhouette coefficient is naturally the optimal number of clusters.

(2) Segmentation method of indicators

The segmentation method of indicators can be briefly described as consisting of four steps:

① Based on the original data, calculate the specific values of the four indicators for all customers, namely Ri, Fi, Mi, and Qi.

② According to the scores of the RFMQ indicators of all customers, divide the indicator values into N segments.

③ Based on the score of each customer, incorporate the customers into the corresponding segmented intervals one by one.

④ According to the actual application situation, further segment the customers that fall into different segmented intervals.

#### 3.2.1 Customer segmentation based on K-means algorithm

**Figure 1:**
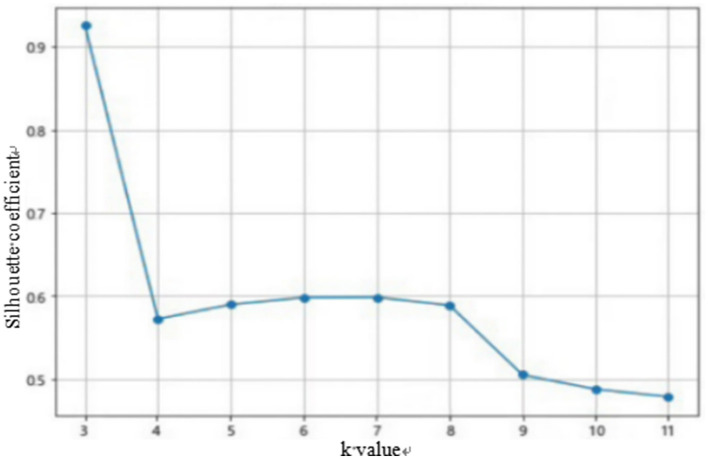
Elbow rule diagram.

**Figure 2:**
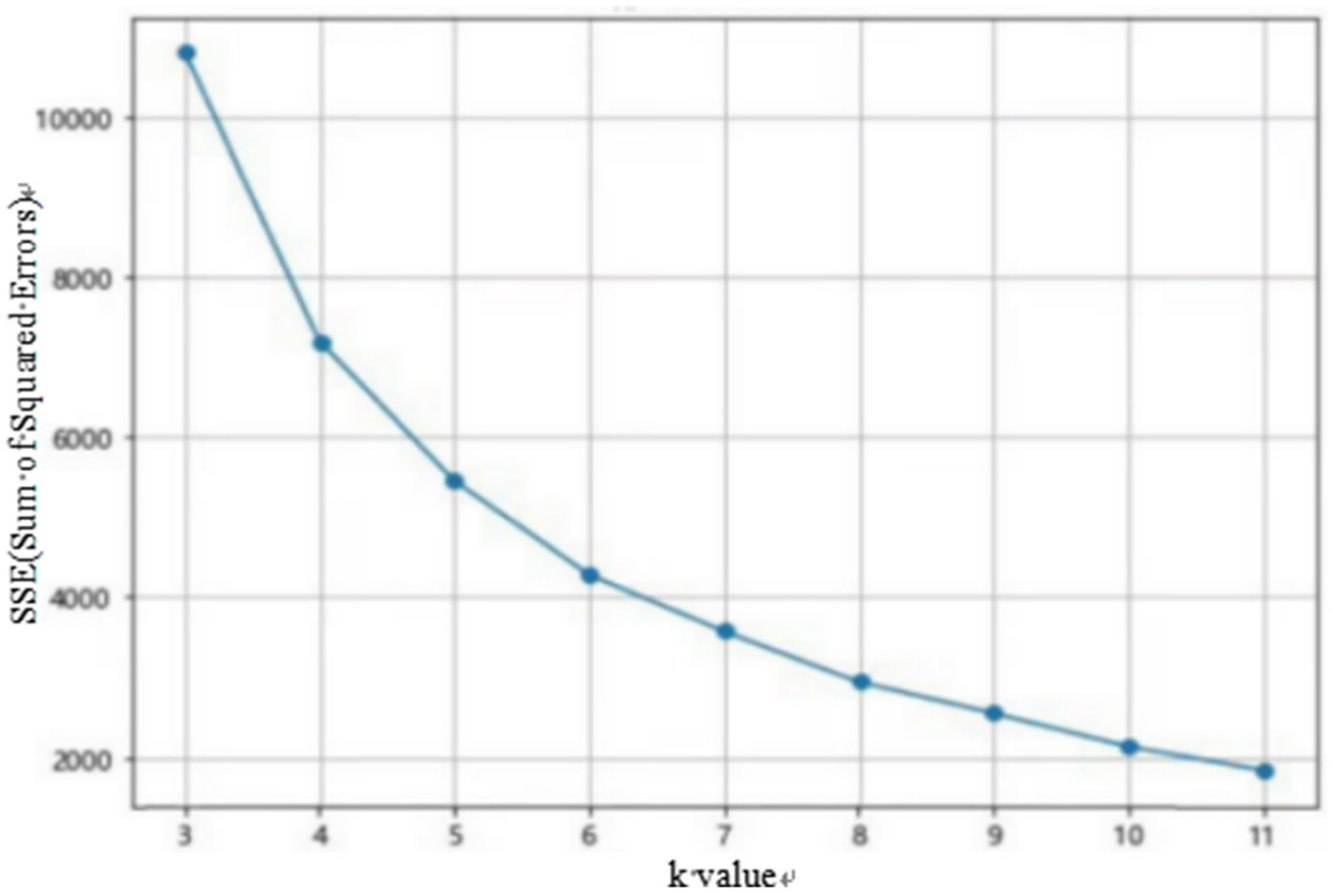
Profile coefficient method diagram.

Before performing K-means clustering, Z-score standardization must also be carried out. This process will not be elaborated here. After standardizing the data, the optimal number of clusters K needs to be determined. Common methods include the elbow rule and the silhouette coefficient method. This paper uses Anaconda's Spyder for calculations. The calculation results of the elbow rule and the silhouette coefficient method are shown in Figures 1, 2.

From the elbow rule graph in Figure 1, it can be seen that when the number of clusters K is 3, the curvature corresponding to the elbow point is the highest. From the silhouette coefficient graph in Figure 2, it can be intuitively observed that when the number of clusters K is equal to 3, the average silhouette coefficient is the largest. Based on this, this paper uses the K-means clustering algorithm to divide all customers of the platform into three categories: high-value customers, general-value customers, and low-value customers. The specific output results are shown in Table 2 below.

**Table 2:** K-means customer segmentation details.

**Customer category**	**R-mean**	**F-mean**	**M-mean**	**Q-mean**	**Number**	**Percentage**
Low-value customers	−1.529	−0.352	−0.161	−0.176	3,203	74.25
General value customers	0.517	0.071	−0.014	−0.009	1,092	25.31
High-value customers	0.693	8.207	11.578	11.702	19	0.44

As can be seen from the results of Table 2 on the customer category segmentation, by using the K-means clustering algorithm to segment all customers, the optimal number of clusters was determined to be 3 using the elbow rule and the silhouette coefficient method, which divided all customers into three categories. Based on the specific values of the R, F, M, and Q indicators of these three categories of customers and the relationship between the within-class means and the overall means of the R, F, M, and Q indicators of all customers, these three categories of customers were defined as high-value customers, general-value customers, and low-value customers, respectively. There are a total of 19 high-value customers, accounting for 0.44% of the total number of customers, which is relatively small. The characteristics of this type of customer include shorter consumption intervals, higher consumption frequencies, larger consumption amounts, and more purchase quantities. There are 1,092 general-value customers, accounting for 25.31% of the total number of customers. The characteristics of this type of customer are that the consumption intervals, consumption frequencies, consumption amounts, and purchase quantities are all at a medium level, and they have relatively stable consumption behaviors. There are 3,203 low-value customers, accounting for 74.25% of the total number of customers. The consumption behavior of this type of customer is relatively weak, with longer consumption intervals, lower consumption frequencies, smaller consumption amounts, and purchase quantities. However, the customer segmentation conducted in this paper is also for the preparation of subsequent cluster recommendations. Based on the K-means RFMQ model's customer segmentation results, the number differences among each customer group are large. Among the high-value customers, there are only 19, which is not particularly suitable for cluster recommendations. Next, the most commonly used segmentation method in practice will be used to classify customers.

#### 3.2.2 Customer segmentation based on indicator segmentation method

During the data preprocessing stage, this paper has standardized the original data to eliminate the dimensional differences among different indicators and ensure the comparability of the data. On this basis, the indicator segmentation method will be further utilized to further segment the customers. The idea of the indicator segmentation method is to allocate the scores of customers on each standardized indicator to different intervals, thereby distinguishing different types of customers.

First, the segmentation standards for each indicator will be set. Common segmentation standards include the quartile method and the equidistant segmentation method. To make the customer groups have more obvious differences, this study adopts the quartile method for segmentation. The quartile method divides customers into four categories based on their standardized scores on each indicator: low, belonging to the lowest 25% of customers; medium-low, belonging to 25%−50% of customers; medium-high, belonging to 50%−75% of customers; high: belonging to the highest 25% of customers.

After the segmentation standards are determined, the standardized scores of customers on each indicator will be divided successively. When segmenting the R value, customers with lower scores (i.e., the most recent purchasers) will be classified as high-active customers; while customers with higher scores (i.e., those who haven't purchased for a long time) will be classified as low-active customers. When segmenting the F value, customers with higher purchase frequency will be classified as high-frequency customers, and those with lower purchase frequency will be classified as low-frequency customers. When segmenting the M value, customers with higher consumption amounts will be classified as high-consumption customers, and those with lower consumption amounts will be classified as low-consumption customers. When segmenting the Q value, customers with more purchased items will be classified as high-purchase quantity customers, and those with fewer purchased items will be classified as low-purchase quantity customers. After segmentation, each customer is assigned to the corresponding interval on each indicator, and a label consisting of four dimensions is created for each customer. Based on these labels, customers can be classified into different groups, namely the high-value customer group, which refers to customers whose standardized scores on both the R and M indicators are above the 25th percentile of all customers; the potential customer group, which refers to customers with a low R value, indicating that they have made purchases recently and have moderate F and Q values; the low-frequency customer group, which refers to customers with the lowest F score among the three values and generally moderate other values; and the churn customer group, which refers to customers with low scores in all three values. After segmentation, the segmented results for each customer in the four dimensions of R value, F value, M value, and Q value can be obtained. Some of the results are shown in Table 3.

**Table 3:** Results of customer segmentation based on indicator segmentation method.

**CustomerID**	**Recency**	**Frequency**	**Monetary**	**Quantity**	**Recency_ Segment**	**Frequency_ Segment**	**Monetary_ Segment**	**Quantity_ Segment**
14259	−0.489511655	−0.42786559	−0.20936681	−0.218819514	Medium-low	Low	Low	Low
14261	0.416794578	−0.29513064	−0.08556341	−0.150945094	Medium-low	Medium-low	Medium-high	Medium-high
14262	0.845049172	0.50127903	0.090853614	0.114477409	High	High	High	High
14264	−0.618983974	−0.29513064	−0.180299512	−0.215467691	Low	Medium-low	Medium-low	Low
14265	−0.160851153	−0.29513064	−0.164664874	−0.17377939	Medium-low	Medium-low	Medium-low	Medium-low
14267	−0.698659247	−0.1623957	−0.046261366	−0.122873574	Low	Medium-high	Medium-high	Medium-high
14270	−2.381799395	−0.42786559	−0.161463399	−0.188862594	Low	Low	Medium-low	Medium-low
14271	−1.326102024	−0.42786559	−0.21023573	−0.217981559	Low	Low	Low	Low
14272	0.187728168	−0.29513064	−0.176150771	−0.149059693	Medium-low	Medium-low	Medium-low	Medium-high
14273	0.416794578	−0.02966075	−0.174655088	−0.190957484	Medium-low	Medium-high	Medium-low	Medium-low

From Table 3, it can be seen the behavioral characteristics of customers in various dimensions. For instance, customer 14,259 belongs to the customer group with medium-low activity level, low frequency of purchase, low consumption, and low purchase volume. While customer 14,262 is a typical customer with high activity level, high frequency of purchase, high consumption, and high purchase volume.

Based on the above segmentation results, customers can be divided into different groups. This article classifies the customers and some of the results are shown in Table 4.

**Table 4:** Customer classification results.

**Cus.tomerID**	**Recency_ Segment**	**Frequency_Segment**	**Monetary_Segment**	**Quantity_Segment**	**Customer_Group**
14259	Medium-low	Low	Low	Low	Low-frequency customers
14261	Medium-low	Medium-low	Medium-high	Medium-high	Low-frequency customers
14262	High	High	High	High	High-value customers
14264	Low	Medium-low	Medium-low	Low	Low-frequency customers
14265	Medium-low	Medium-low	Medium-low	Medium-low	Low-frequency customers
14267	Low	Medium-high	Medium-high	Medium-high	Low-frequency customers
14270	Low	Low	Medium-low	Medium-low	Low-frequency customers
14271	Low	Low	Low	Low	Loss of customers
14272	Medium-low	Medium-low	Medium-low	Medium-high	Low-frequency customers
14273	Medium-low	Medium-high	Medium-low	Medium-low	Low-frequency customers

**Figure 3:**
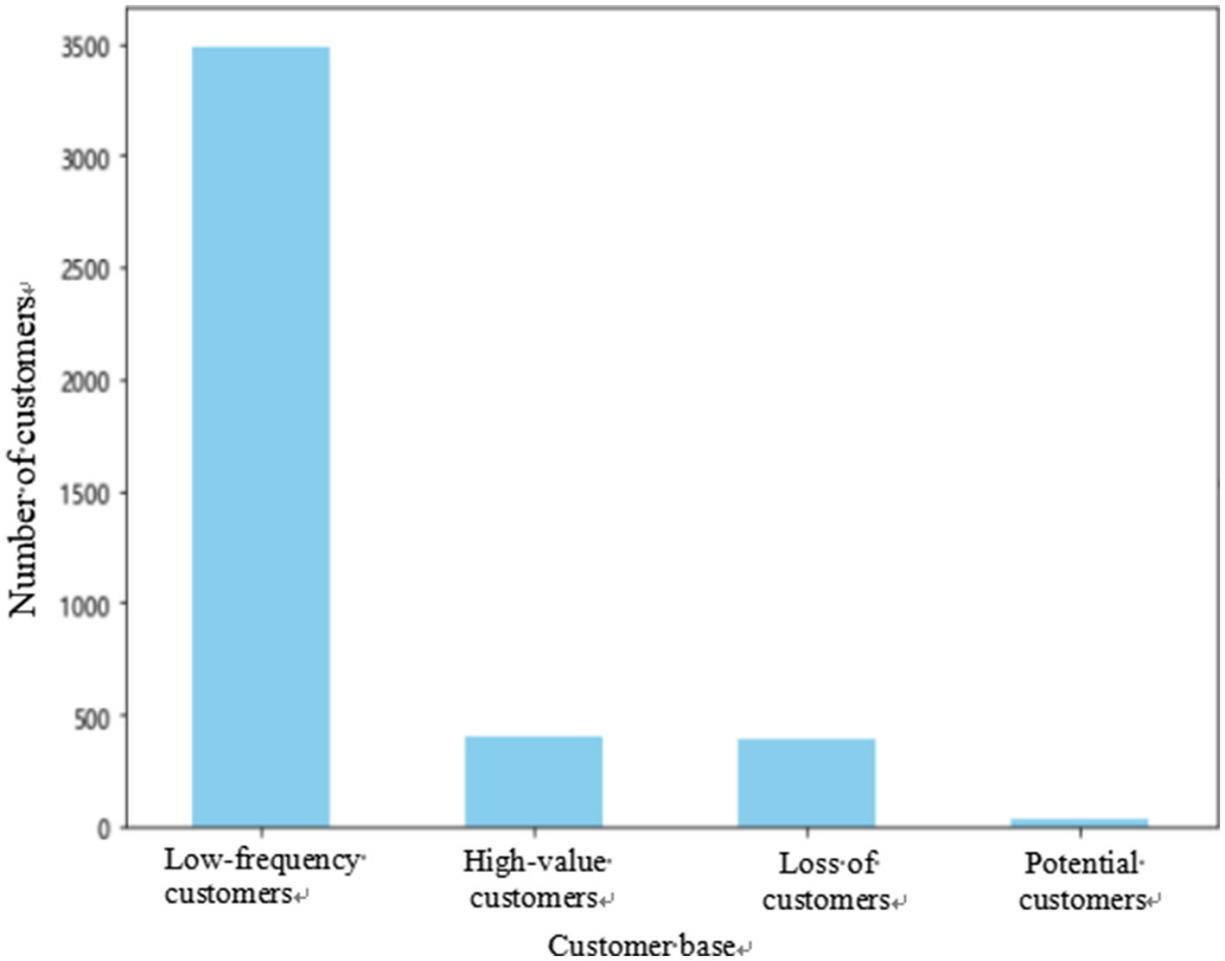
Distribution map of customer groups.

The customer segmentation statistics result of the RFMQ model based on indicator segmentation is shown in Figure 3. Through analysis, on this platform, among a total of 4,314 customers, the segmented groups are distributed as 3,485 low-frequency customers, 401 high-value customers, 393 churned customers, and 35 potential customers.

Using the indicator segmentation method, this study divided the customer groups in the RFMQ model into four categories. Low-frequency customers accounted for 80.78% of the total number of customers on the platform; high-value customers accounted for 9.30% of the total customers; churned customers accounted for 9.11% of the total customers; and potential customers accounted for 0.81% of the total customers. From the distribution results, the proportion of low-frequency customers was the largest, which generally aligns with the actual situation. Although the proportion of high-value customers in the group was small, they contributed significantly to the platform. Therefore, this group needs to be given more attention for maintenance. The proportion of churned customers indicates that some customers may no longer be active, which probably requires merchants to pay attention and take effective measures to recover. The proportion of potential customers was the smallest, but they might be the core customer group for the platform's future development. Overall, this classification method had 401 high-value customers, which is more in line with the actual situation compared to the customer segmentation using the K-means clustering algorithm, and is convenient for subsequent personalized recommendations based on group segmentation.

### 3.3 Data analysis

#### 3.3.1 Data sources and processing

By establishing the RFMQ model and using the index segmentation method and K-means clustering analysis method for customer segmentation, we obtained the corresponding customer group data. Next, this paper uses the customer group data obtained from the above methods to carry out collaborative filtering recommendation experiments.

This study employs a user-centric collaborative filtering recommendation algorithm, which requires evaluating customers' preference levels for purchased items through their product reviews. The original data used consists solely of historical transaction records from platform merchants, where customer preferences can only be indirectly inferred based on displayed ratings. In traditional user-collaborative filtering recommendation algorithms, 0-1 matrices are typically employed to represent customer-product interactions, where 1 indicates purchase and 0 signifies no purchase. This method has inherent limitations: when multiple identical products are purchased by one customer while another buys only one item, the traditional 0-1 matrix equates both customers' preferences, failing to accurately reflect actual purchasing behavior. To address this issue, this paper develops a more precise approach by analyzing prior research and literature. We first extract four key metrics—R, F, M, and Q—from customers' purchase histories, then calculate their weights through standardized operations. Ultimately, we derive a weighted average rating that establishes a comprehensive and objective evaluation matrix.

#### 3.3.2 Constructing the customer-product evaluation matrix

(1) Data processing

Before starting the construction of the matrix, the original data need to be preprocessed and standardized using Z-score. An additional product dimension is added. This stage will not be elaborated further. Some results are shown in Table 5.

**Table 5:** Preprocessing results of customer product matrix.

**CustomerID**	**StockCode**	**R**	**F**	**M**	**Q**	**R_scaled**	**F_scaled**	**M_scaled**	**Q_scaled**
14578	85225	2	1	1	4	−1.19138	−0.3499	−0.39677	−0.06375
14578	90093	2	1	0.19	20	−1.19138	−0.3499	−0.49558	0.002286
14581	21034	16	1	0.95	1	−1.06545	−0.3499	−0.40287	−0.07613
14581	22573	16	1	0.85	5	−1.06545	−0.3499	−0.41507	−0.05962
14581	22574	16	1	0.85	6	−1.06545	−0.3499	−0.41507	−0.0555
14581	22610	16	1	0.19	36	−1.06545	−0.3499	−0.49558	0.068323
14581	22731	16	2	2.5	11	−1.06545	0.394371	−0.21379	−0.03486
14581	22732	16	1	1.25	9	−1.06545	−0.3499	−0.36627	−0.04311
14581	22734	16	1	2.89	5	−1.06545	−0.3499	−0.16621	−0.05962
14581	22910	16	1	2.95	1	−1.06545	−0.3499	−0.15889	−0.07613

(2) Calculation of index weights

To objectively calculate the weights of each index, this paper uses the entropy method to calculate the weights of the four indicators R, F, M, and Q. The main advantage of the entropy method is that it can automatically calculate the weights based on the data distribution of each index, thereby avoiding the subjective deviations that may occur when setting the weights manually. The core idea of this method is to measure the uncertainty degree of each indicator by calculating its information entropy. In this case, the larger the information entropy, the more abundant the information contained in the indicator, and the higher the corresponding weight will be.

As early as 1948, Wiener and Shannon proposed the concept of information entropy. They first introduced the concept of entropy from thermodynamics into information theory. Information entropy refers to the uncertainty of information. Information entropy uses probability testing and data statistics to study the degree of uncertainty of the signal states output by the information source during the communication process. Therefore, information entropy can be used to measure the uncertainty of the system state represented by probability, that is, if a system has n different states and the probability of each state is pi, then the entropy of this system is:


(4) $$\begin{array}{l} E\;=\;-\sum_{i=1}^{n}p_{i}\ln\;p_{i}Amongthem,p_{i}content\;with:\\ \;0\leq p_{i}\leq 1;\;\sum_{l=1}^{n}p_{i}=1 \end{array}$$

There exists a multi-feature dataset consisting of m random variables and n features. After normalizing the data, a matrix R is obtained.


$$\begin{array}{l} R=\left[\begin{array}{cccc} r_{11} & r_{12} & \cdots & r_{1n}\\ r_{21} & r_{22} & \cdots & r_{2n}\\ \cdots & \cdots & \ddots & r_{m1}\\ r_{m1} & r_{m2} & \cdots & r_{mn} \end{array}\right] \end{array}$$

In matrix R, rij represents the specific value under a certain feature. The detailed calculation method for the weights of each feature is given in Equation 5.


(5) $$\begin{array}{l} w_{j}=\frac{1-E_{j}}{\sum_{j=1}^{n}\left(1-E_{j}\right)} \end{array}$$

Among them,


(6) $$\begin{array}{l} E_{j}=-\frac{1}{\ln\;m}\sum_{i=1}^{m}\frac{r_{ij}}{\sum_{i=1}^{m}r_{ij}}\ln\frac{r_{ij}}{\sum_{i=1}^{m}r_{ij}} \end{array}$$

Obviously, we have $\overset{n}{\underset{j=1}{\sum }}w_{j}=$ 1, and 0 ≤ *w*_*j*_ ≤ 1. Here, Ej is the extended form of information entropy, which is also the reason for the naming of the entropy value method.

This paper uses Python 3.9 to calculate the weight values of each indicator by using the entropy method. The following steps are adopted: calculate the proportion of each indicator's data points to the total sum of the indicators; based on each proportion value, calculate the information entropy and determine the uncertainty level of each indicator; obtain the entropy weight by subtracting the normalized value of the information entropy from 1, ensuring that the weights of each indicator can correctly reflect their relative importance. The weights of each indicator obtained after processing are as shown in Table 6.

**Table 6:** Results of index weighting.

**Metric**	**Weight**
R	0.078993
F	0.389981
M	0.145359
Q	0.385667

So the final weights for R, F, M, and Q are determined to be 0.079, 0.390, 0.145, and 0.386, respectively. That is (WR, WF, WM, WQ) = (0.079, 0.390, 0.145, 0.386).

(3) Construction of customer product rating matrix

Here, first, standardized data is obtained based on Z-score and combined with the weights of R, F, M, and Q indicators. The customer ratings for the products are calculated through the weighted average method. The data format for indicator standardization and rating calculation is as shown in Table 7.

**Table 7:** Standardization of indicators and calculation of scores.

**CustomerID**	**StockCode**	**R**	**F**	**M**	**Q**	**R_scaled**	**F_scaled**	**M_scaled**	**Q_scaled**	**rating**
1	1	R1	F1	M1	Q1	R'1	F'1	M'1	Q'1	Ra1
2	2	R2	F2	M2	Q2	R'2	F'2	M'2	Q'2	Ra2
…	…	…	…	…	…	…	…	…	…	…
m	n	Rm	Fm	Mm	Qm	R'm	F'm	M'm	Q'm	Ram

The columns R_scaled, F_scaled, M_scaled, and Q_scaled in the table represent the standardized values of R, F, M, and Q, respectively. The final standardized values of each indicator of the rating are the weighted average of these values. As known from the previous step, the weights of the four indicators R, F, M, and Q are 0.079, 0.390, 0.145, and 0.386, respectively. The calculation formula for the score is as shown in Equation 7:


(7) $$\begin{array}{l} \text{Rating}=0.079\text{R\_scaled}+0.390\text{F\_scaled}\\ \;+0.145\text{M\_scaled}+0.386\text{Q\_scaled} \end{array}$$

After going through the above process, the customer's rating of the product is obtained, and the customer's rating table of the product can be obtained. The example is shown in Table 8 below. Based on this, a customer-product rating matrix is constructed. The rules for converting the rating matrix: ① The rows of the matrix are the products, and the columns are the customers. ② The scores Rating generated by the customers for the products they have transacted are filled in the matrix. ③ For the products that the customers have not transacted, the rating value is empty. The output result example is shown in Tables 8, 9 below:

**Table 8:** Customer product rating results.

**CustomerID**	**StockCode**	**Rating**
14578	85225	−0.31288
14578	90093	−0.30178
14581	21034	−0.30859
14581	22573	−0.30399
14581	22574	−0.3024
14581	22610	−0.26636
14581	22731	0.025055
14581	22732	−0.29053
14581	22734	−0.26782
14581	22910	−0.27312

**Table 9:** Customer product rating matrix results.

**StockCode**	**14,578**	**…**	**14,583**	**…**	**14,587**	**…**	**14,591**	**…**	**14,593**	**…**	**14,595**	**14,597**	**…**	**14,603**	**14,606**	**…**	**14,609**
…																	
10135															0.143094		
…																	
15036														0.187998			
…																	
16048									−0.31975								
…																	
16216			−0.19787														
16218			−0.19787				−0.27964										
…																	
16235							−0.30881								0.596411		
16236												−0.32598					

(4) Search for nearest neighbors

Based on the previous customer segmentation and the customer product rating matrix, this section searches for the nearest neighbor set for the target customer among the various value groups of the customers on the e-commerce platform. The specific steps are as follows: ① Determine the group category of the target customer based on its CustomerID. ② Select all other customers who belong to the same customer group as the target customer. ③ Calculate the similarity between the target customer and other customers in the same customer group using the rating values of each customer in the customer product rating matrix. This article uses cosine similarity, and the specific calculation method is shown in Equation 8 below. ④ Select the K nearest neighbors with the highest similarity to the target customer.

The calculation method of cosine similarity is relatively simple. It first maps the customer's product ratings to the Euclidean space to obtain two vectors, then calculates the cosine value of the angle between these two vectors, and finally uses the magnitude of the cosine value to reflect the similarity between customers. The specific calculation method is as shown in Equation 8:


(8) $$\begin{array}{l} \begin{array}{cc} sim\left(u_{1},u_{2}\right) & =\cos\left(u_{1},u_{2}\right)=\frac{\sum_{i\in R_{U}}r_{i1}r_{i2,1}}{\sqrt{\sum_{i\in R_{U}}r_{i1}^{2}\sum_{i\in R_{U}}r_{i2,1}^{2}}} \end{array} \end{array}$$

Among them, Ru represents the set of items that both customers and have rated together.

#### 3.3.3 Collaborative filtering recommendation based on customer segmentation

The idea of the recommended method is to generate TOPN recommendations through the constructed customer-product matrix, combined with customer segmentation (segmentation by indicators and K-means clustering analysis method). The specific method has been described earlier, and will not be elaborated further here. Taking customer 14,578 as an example, 10 products are recommended. The two recommendation results are as follows. The result of the segmentation by indicators is shown in Table 10.

**Table 10:** Recommended results based on segmentation of indicators.

**The products recommended to the customers 14,578**		
**StockCode**	**Predictive scoring**	**Product**
22424	0.430162	Basketball training auxiliary marking disc
23110	0.186689	Football tactics board
22960	0.143044	Badminton racket set
23153	0.118944	Table tennis net frame
20819	0.116417	Running heart rate monitoring wristband
22158	0.103307	Jump Rope (Speed racing version)
22558	0.069938	Yoga mat (anti-slip version)
21588	0.067493	Plank support
22644	0.062846	Mountaineering stick (aluminum alloy)
20622	0.059493	Knee pads (professional sports model)

The results of the K-means clustering method are presented in Table 11.

**Table 11:** Recommended results of K-means clustering method.

**The products recommended to the customers 14,578**		
**StockCode**	**Predictive scoring**	**Product**
20819	0.116417	Running heart rate monitoring wristband
21588	0.067493	Plank support
20622	0.059493	Knee pads (professional sports model)
48194	0.027309	Basketball (Standard size 7)
48138	0.027309	Football (Standard size 5)
22688	0.027309	Badminton (Resilient model)
22366	0.027309	Table tennis (Three-star quality)
22837	−0.02962	Swimming earplug nose pad set
20818	−0.04557	Riding helmet (Breathable model)
22423	−0.06006	Fitness pulling rope set

Among them, in the prediction score, a score greater than 0 indicates that the customer has a high interest in the product. A score close to 0 suggests that the customer has a low interest in the product, but still has a certain possibility of purchasing intention. A score less than 0 indicates that the customer is unlikely to purchase the product.

#### 3.3.4 Traditional collaborative filtering recommendation

For comparison, the following describes traditional collaborative filtering methods. These approaches typically use a 0-1 matrix to represent customer-item interactions. This study employs such a matrix as the customer-item matrix (where 0 indicates no purchase and 1 indicates a purchase), which is then used for collaborative filtering recommendations. Partial results of this matrix are shown in Table 12.

**Table 12:** Results of 0-1 matrix.

**CustomerID**	**71,053**	**22,752**	**21,730**	**22,633**	**22,632**	**84,879**	**22,745**	**22,748**	**22,749**
17850	1	1	1	1	1	0	0	0	0
13047	0	0	0	0	0	1	1	1	1
12583	0	0	0	0	0	0	0	0	0
13748	0	0	0	0	0	0	0	0	0
15100	0	0	0	0	0	0	0	0	0
15291	0	0	0	0	0	1	0	0	0
14688	0	0	0	0	0	0	0	0	0
17809	0	0	0	0	0	0	0	0	1
15311	0	1	0	0	0	0	0	0	0
16098	0	0	0	0	0	0	0	0	0
18074	0	0	0	0	0	0	0	0	0
17420	0	0	0	0	0	0	0	0	0
16029	0	0	0	0	0	0	0	0	0
16250	0	0	0	0	0	0	0	0	0
12431	0	0	0	0	0	0	0	0	0

After the collaborative filtering recommendation, the results of recommending the goods to customer 14,578 are shown in Table 13.

**Table 13:** Collaborative filtering recommendation results.

**The 14,578 product recommended to the customer**		
**StockCode**	**Recommendation strength**	**Product**
21479	2	Basketball (for indoor and outdoor use)
23357	2	Football (training model)
23356	2	Badminton (competition grade)
23439	2	Table tennis (competition grade)
23076	1	Tennis (training model) Ski goggles
22866	1	Boxing gloves (beginner model)
23118	1	Retro spotted pattern grip exerciser Football tactics board
21485	1	Baseball bat (aluminum alloy)
23110	1	Basketball (for indoor and outdoor use)
82482	1	Football (training model)

Among them, the recommendation strength indicates the number of similar customers who have purchased this product. The higher the value, the more times similar customers have purchased this product, and the more worthy it is to be recommended.

#### 3.3.5 Experimental method

The experimental environment is based on the Windows 64-bit operating system, primarily utilizing Anaconda's Spyder (Python 3.9) for operations. Given the difficulty in obtaining real purchase records after recommendation results, this study employs fivefold cross-validation (5-fold cross-validation) as the model evaluation method. This approach divides the dataset into five subsets, selecting four for model training and one for testing each time. The process is repeated five times, with each subset serving as a test set once. Ultimately, the overall performance of the model is assessed by calculating the average results from all five tests, thereby enhancing experimental stability and result reliability. The experimental metrics are categorized into two aspects.

(1) Customer satisfaction

Customer satisfaction is a measure of customers' subjective feelings about recommendation results, reflecting their recognition and acceptance of recommended products. This indicator can be obtained through questionnaires, interviews, or behavioral data. However, due to the subjectivity of customer satisfaction and the difficulty in data collection, this metric is rarely used as a mainstream evaluation standard.

(2) Accuracy index

The TOPN recommendation method employed in this study operates as a classification prediction model. It first calculates customer similarity to identify customers with high relevance to the target customer, thereby constructing a similarity matrix. Recommendations are ultimately generated based on these similarities, resulting in a TOPN recommendation list. Key performance metrics in implementing this method include Precision (accuracy), Recall (hit rate), and F1 value.

Precision (Precision) measures the proportion of the products that customers actually like among all the recommended products. The formula is:


(9) $$\begin{array}{l} \begin{array}{c} Precision=\frac{TP}{TP+FP}\; \end{array} \end{array}$$

Among them, TP refers to the positive result and the predicted result, and FP refers to the negative result and the predicted result.

The recall rate (Recall) measures the proportion of the system's recommended products among all the actual positive products that customer actually like. The formula is:


(10) $$\begin{array}{l} \begin{array}{c} Recall=\frac{TP}{TP+FN}\; \end{array} \end{array}$$

Among them, FN refers to the real result is positive and the predicted result is negative.

F1 score is the harmonic average of precision and recall, which comprehensively considers the performance of these two indicators. It is a comprehensive index for recommendation algorithm evaluation. The formula is:


(11) $$\begin{array}{l} \begin{array}{c} F1\;score=\frac{2}{\left(\frac{1}{Precison}+\frac{1}{Recall}\right)}=\frac{2*Precison*Recall}{Precision+Recall}\; \end{array} \end{array}$$

In the aforementioned evaluation metrics, customer satisfaction measurement typically relies on feedback regarding recommendation outcomes, though such feedback is not easily obtained. Furthermore, as this study employs the TOPN recommendation method, accuracy, recall rate, and F1 value were selected as evaluation indicators to assess recommendation effectiveness. These metrics effectively measure the quality of recommendations from a customer's perspective.

## 4 Results and discussion

Based on the collected data, three experimental methods will be compared for recommendation performance evaluation. These methods include traditional collaborative filtering and two customer segmentation-based approaches (segmentation through metric segmentation and K-means clustering), both utilizing the RFMQ model as their foundational framework. The evaluation metrics consist of precision, recall, and F1 score obtained from each experiment.

(1) Evaluation results based on traditional collaborative filtering recommendation

As mentioned earlier, the traditional user-based collaborative filtering recommendation algorithm usually uses a 0-1 matrix to represent the evaluation between customers and products. In this paper, this method is adopted as the matrix and then used for recommendation. The evaluation effect of traditional collaborative filtering recommendation is shown in Table 14.

**Table 14:** Effectiveness of traditional collaborative filtering recommendation.

**Evaluation value**	**TOPN = 3**	**TOPN = 5**	**TOPN = 7**	**TOPN = 10**
Precision	0.3104	0.3923	0.2814	0.2707
Recall	0.0435	0.0575	0.0866	0.1823
F1 score	0.1624	0.1709	0.1745	0.1759

**Figure 4:**
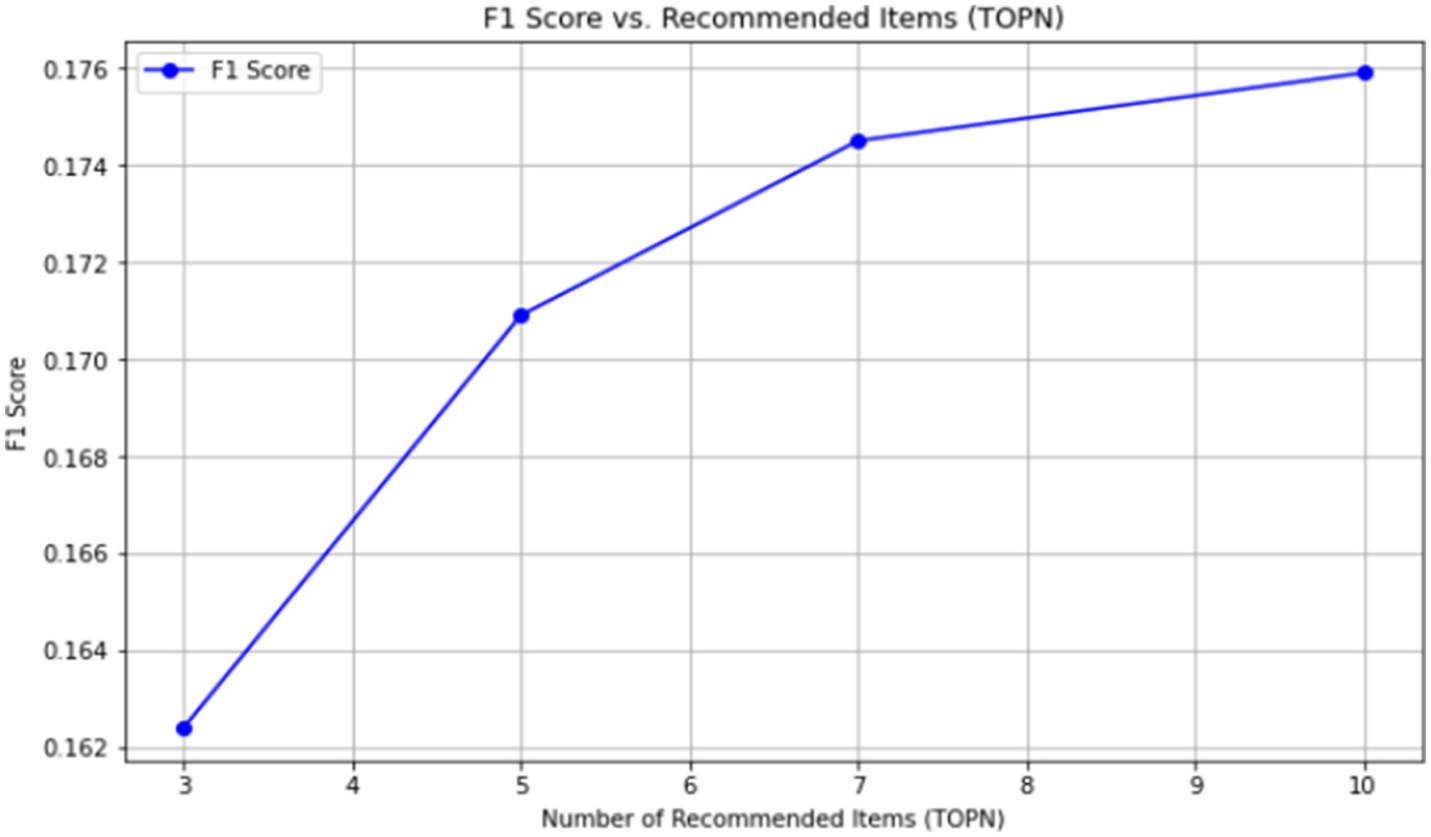
Line graph of F1 value for evaluation.

The line graph of its F1 value is shown in Figure 4.

Table 14 shows that the TOP3 recall rate of the traditional collaborative filtering recommendation effect is only 0.0435, the TOP5 recall rate is only 0.0575, the TOP7 recall rate is only 0.0866, and the TOP10 recall rate is 0.1823. The recall rate increases successively. The reason for the low recall rate is often caused by the data sparsity in the customer product matrix. Especially when customer segmentation has not been conducted, the data set used in this paper has a large number of low-value customers. Without performing classification-based collaborative filtering recommendation, data sparsity will inevitably occur, and the interaction behavior of customers with products is also relatively less.

(2) Evaluation results of traditional collaborative filtering recommendation

Evaluation results of collaborative filtering recommendation model based on index division method in RFMQ.

The recommendation effect of each customer group is shown in Table 15.

**Table 15:** Collaborative filtering recommendation effect based on index classification method.

**Customer base**	**Evaluation indicators**	**TOPN = 3**	**TOPN = 5**	**TOPN = 7**	**TOPN = 10**
High value customers	Precision	0.7238	0.6482	0.4719	0.3794
	Recall	0.3312	0.1528	0.0685	0.0213
	F1 score	0.5969	0.5508	0.3672	0.1814
Low-frequency customers	Precision	0.4129	0.3487	0.2834	0.1385
	Recall	0.2084	0.1915	0.0793	0.0217
	F1 Score	0.4061	0.3234	0.2767	0.1378
Customer churn	Precision	0.3592	0.2235	0.1037	0.0384
	Recall	0.2638	0.2084	0.1015	0.0392
	F1 score	0.2604	0.2201	0.0987	0.0428
Potential customers	Precision	0.2914	0.2138	0.1682	0.1327
	Recall	0.1919	0.1423	0.0096	0.0113
		F1 score	0.1872	0.1370	0.0069	0.0099

The average F1 values are shown in Table 16.

**Table 16:** Results of the recommended effect average F1 value based on the indicator classification method.

**TOPN**	**3**	**5**	**7**	**10**
Average F1 score	0.3642	0.3093	0.1989	0.1829

**Figure 5:**
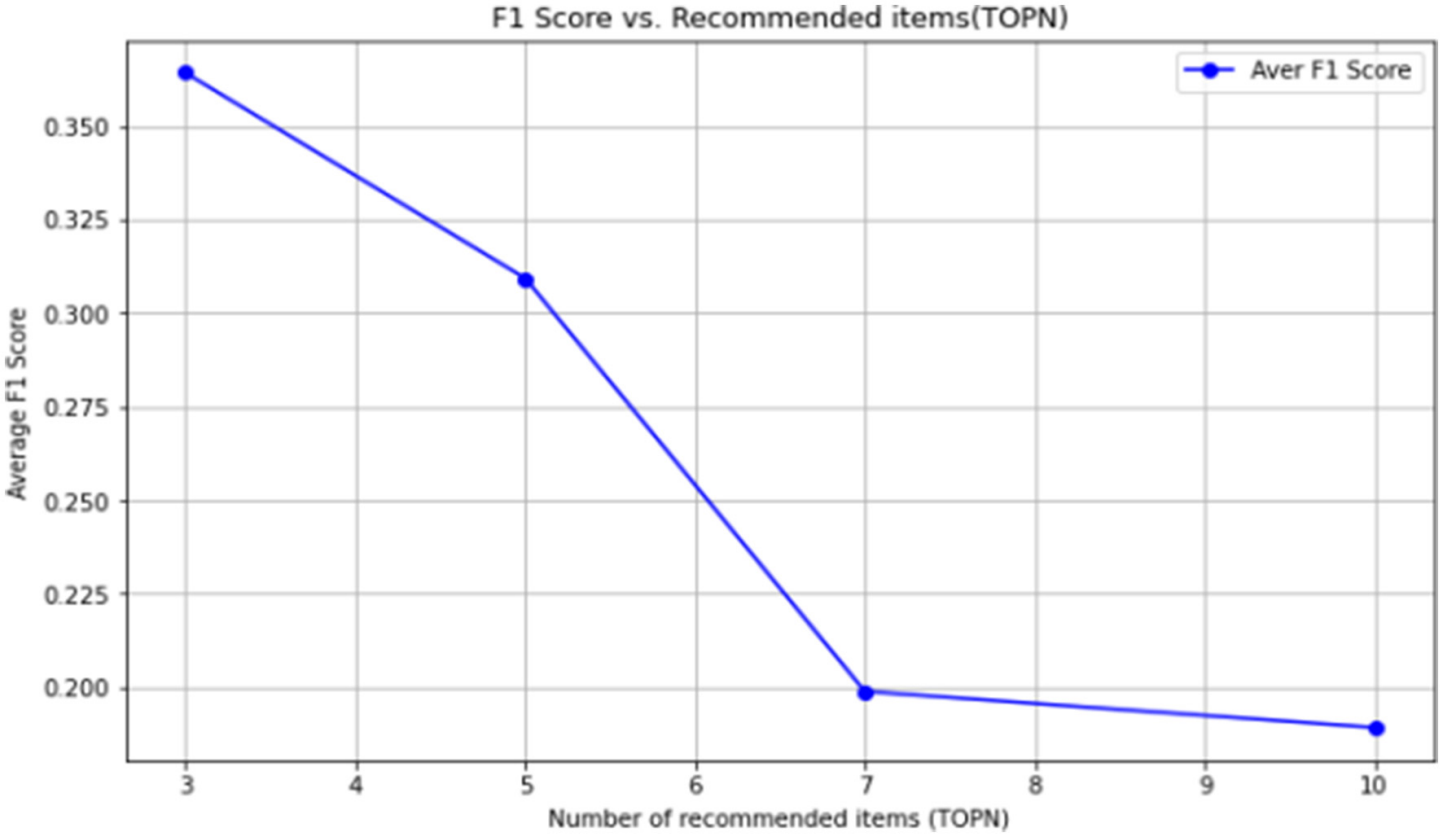
Line chart of average F1 values for evaluation effects of collaborative filtering recommendation by index division.

The line graph is shown in Figure 5.

(3) Evaluation of the recommendation effectiveness of the RFMQ model based on the K-means clustering method

The recommendation evaluation results for the three customer groups are shown in Table 17.

**Table 17:** Results of collaborative filtering recommendation based on K-means clustering method.

**Customer type**	**Evaluating indicator**	**TOP3**	**TOP5**	**TOP7**	**TOP10**
Low-value customers	Precision	0.482	0.421	0.376	0.318
	Recall	0.234	0.181	0.127	0.074
	F1	0.316	0.253	0.188	0.12
High value customers	Precision	0.498	0.463	0.412	0.387
	Recall	0.358	0.294	0.203	0.165
	F1	0.416	0.357	0.272	0.232
The average value customer	Precision	0.341	0.305	0.301	0.302
	Recall	0.162	0.138	0.089	0.063
	F1	0.219	0.191	0.137	0.104

The average F1 value is shown in Table 18.

**Table 18:** Average F1 value results of the K-means clustering method recommendations.

**TOPN**	**3**	**5**	**7**	**10**
Average F1 score	0.317	0.267	0.199	0.185

**Figure 6:**
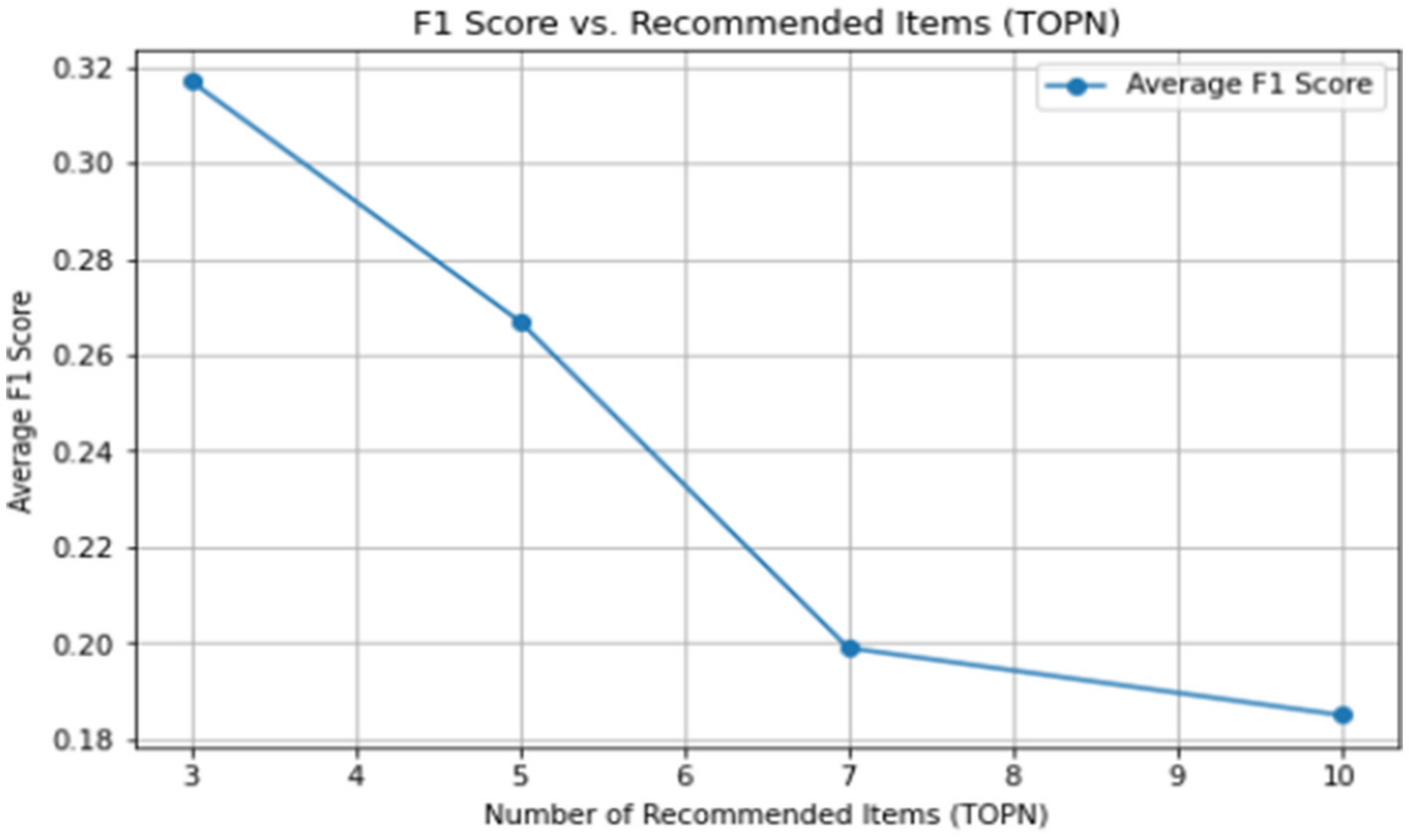
Line graph showing the average F1 values of the evaluation results of the K-means clustering analysis method for collaborative filtering recommendation method results.

The line graph of the average F1 value is shown in Figure 6.

### 4.1 Comparative analysis of recommendation effectiveness

As shown in Tables 14, 15, 17, the recall rates (Recall) of all methods are relatively low. This is in line with the general characteristics of the TOP-N recommendation task: since the length of the recommendation list N is much smaller than the total number of products that the user may like, the system focuses more on the accuracy of recommendations rather than full coverage. The lower recall rate is acceptable in this scenario. The core objective of this study is to ensure the accuracy of the top part of the recommendation list (measured by Precision and F1) and the sorting quality.

**Figure 7:**
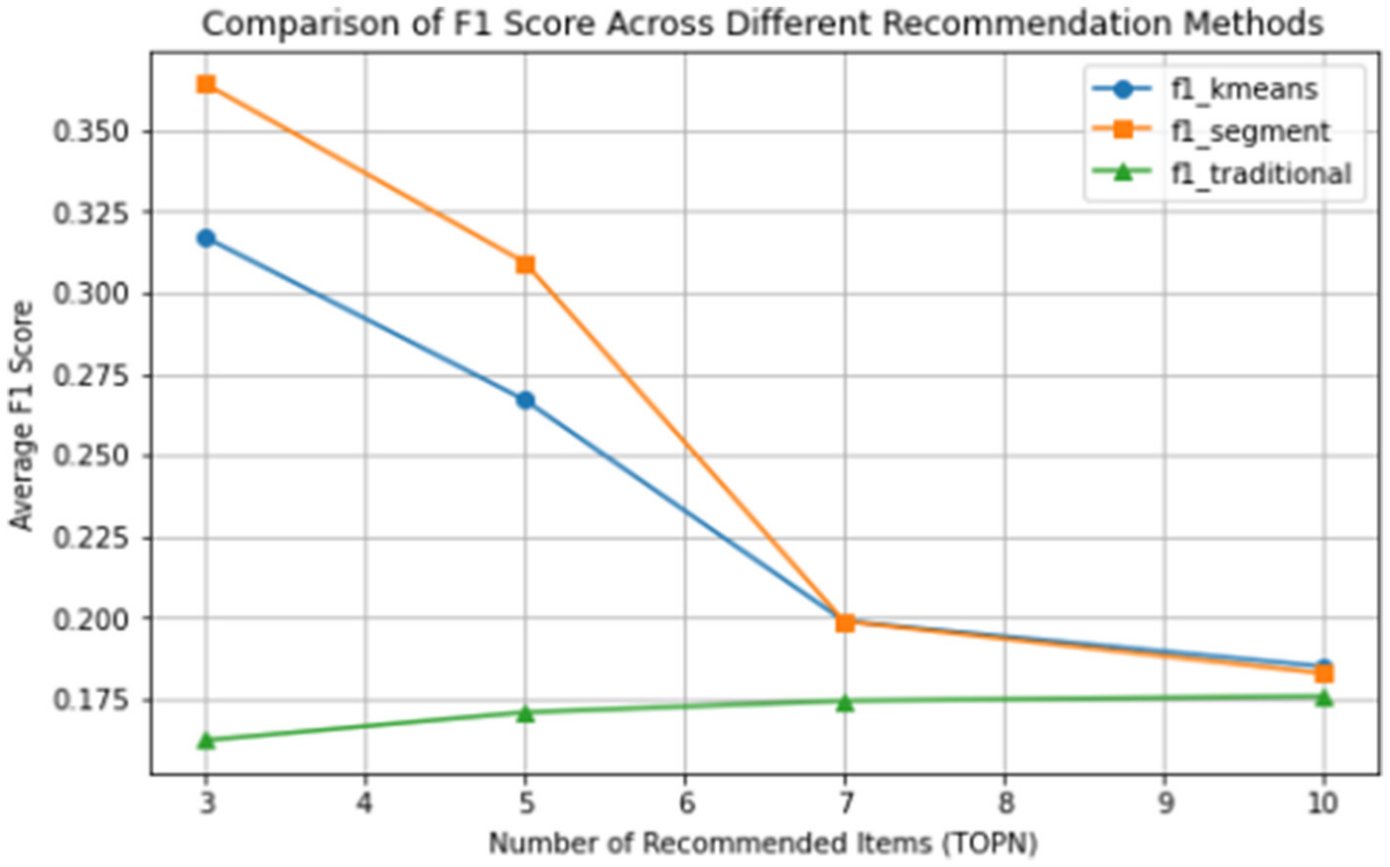
Comparison of F1 curves for the three recommendation methods.

From the line graph comparing the F1 values of different recommendation methods under different numbers of recommended products (i.e., Figure 7), it can be seen that among the three recommendation methods, the traditional collaborative filtering has the poorest effect, the collaborative filtering recommendation based on indicator segmentation has the best effect, and the collaborative filtering recommendation based on the K-means method is in between, but its F1 value is significantly higher than that of the traditional collaborative filtering recommendation.

This shows that in traditional collaborative filtering recommendations, as the number of recommended products increases, the accuracy improves, but the improvement is not obvious. In the collaborative filtering recommendation method based on customer segmentation, the recommendation effect decreases as the number of recommended products increases. When the number of recommended products is between 3 and 5, compared with traditional collaborative filtering, the recommendation effect is significantly enhanced. When the number of recommended products continues to increase, the recommendation effect begins to drop significantly, and when the number of recommended products increases to 10, the recommendation effect drops significantly, and is almost the same as that of the traditional collaborative filtering method.

## 5 Research summary

This study integrates the RFMQ model with collaborative filtering recommendation algorithms to enhance the traditional RFM model for customer segmentation and product recommendation optimization in cross-border e-commerce. By analyzing historical customer transaction data, we developed the RFMQ model. Through multiple segmentation methods to categorize customers into distinct groups, collaborative filtering recommendations were implemented to improve recommendation quality.

Experimental results demonstrate that the RFMQ model-based collaborative filtering recommendation system outperforms traditional methods in various evaluation metrics through customer segmentation. This superiority fundamentally stems from the model's ability to significantly enhance user profile accuracy and behavioral representation richness via multidimensional customer segmentation, thereby systematically optimizing the core mechanisms of collaborative filtering.

The RFMQ model with indicator segmentation demonstrated the best performance in collaborative filtering recommendations, followed by the K-means-based RFMQ model. This may be because indicator segmentation can uncover more detailed user characteristics within customer groups. For instance, when analyzing key customers, indicator segmentation allows deeper segmentation within this group, whereas K-means clustering only divides them into a few categories without further refinement. Indicator segmentation effectively avoids the single-group partitioning issue common in K-means clustering, thereby enhancing personalized recommendation quality. Comparative analysis revealed that with fewer recommended items, the optimized method showed significant improvement over traditional collaborative filtering approaches, enabling more satisfactory and appropriate product recommendations for users.

In practical business applications, this approach provides individual merchants and enterprises in cross-border operations with a simple yet cost-effective marketing solution. Particularly suitable for small and medium-sized enterprises (SMEs) and startups, it offers fresh development perspectives. Merchants can break free from reliance on paid platform recommendation services while deploying their own recommendation systems on self-built websites. This enables personalized customer segmentation and targeted marketing strategies, allowing tailored approaches to different value tiers of customers. Such strategies enhance customer satisfaction and drive purchasing behavior.

Although the model method proposed by this research has achieved good results in the experiment, there are still some shortcomings, mainly in the data representativeness, the diversity and richness of experimental data and the dynamic status of the model. These factors may affect the wide applicability and promotion effect of the model.

The data in this study primarily originates from historical transaction records of merchants. While these datasets provide valuable insights for customer behavior analysis, their representativeness remains limited. Given that the dataset exclusively covers a single merchant, it may not fully reflect the broader cross-border e-commerce market, potentially restricting the model's generalizability and leading to varying effectiveness across different merchant types. Furthermore, while the model is built on transactional data, individual merchants' customer bases exhibit unique consumption patterns and characteristics. This inherent limitation means the model's applicability might be constrained. Transactional data inherently represents static attributes, whereas market environments are dynamic—customer preferences continuously adapt to evolving market trends and demands. This dynamic nature could result in recommendation outcomes failing to keep pace with actual user needs. Future research will expand data sources and enrich data dimensions to better validate and address the limitations identified in this study.

## Data Availability

The original contributions presented in the study are included in the article/supplementary material, further inquiries can be directed to the corresponding authors.

## References

[B1] Beretzky E. Hausmann L. Wölfel T. Zimmermann T. (2022). Signed, Sealed, and Delivered: Unpacking The Cross-Border Parcel Market's Promise. McKinsey & Company. Available online at: https://www.mckinsey.com/industries/travel-logistics-and-infrastructure/our-insights/signed-sealed-and-delivered-unpacking-the-cross-border-parcel-markets-promise

[B2] Chavhan R. Dutta P. Samant N. Kar S. (2025). Data-driven strategic customer segmentation considering cart abandonment behavior: insights from e-grocery delivery platforms. Inf. Sci. 718:122327. 10.1016/j.ins.2025.122327

[B3] Chen P. (2024). Research on music teaching content design based on intelligent algorithm optimization under constructivism theory. Appl. Math. Nonlinear Sci. 9:3469. 10.2478/amns-2024-3469

[B4] Chen W. H. Hsu C. C. Lai Y. A. Liu V. Yeh M. Y. Lin S. D. (2020). Attribute-aware recommender system based on collaborative filtering: survey and classification. Front. Big Data 2:49. 10.3389/fdata.2019.00049 33693372 PMC7931907

[B5] China Insights Consultancy (2023). China Sports and Fitness Products IndusBlue Book. Shanghai.

[B6] Fan J. Pan W. Jiang L. (2014). “An improved collaborative filtering algorithm combining content-based algorithm and user activity,” in https://ieeexplore.ieee.org/xpl/conhome/6731712/proceedin2014 International Conference on Big Data and Smart Computing (BIGCOMP) (Bangkok: IEEE), 88–91.

[B7] Goldberg D. Nichols D. Oki B. M. Terry D. (1992). Using collaborative filtering to weave an information tapestry. Commun. ACM, 35, 61–70. 10.1145/138859.138867

[B8] Han J. H. Lai P. L. (2025). The power of digital nativeness: exploring how millennials mitigate psychic distance in cross-border electronic commerce. Technol. Soc. 82:102917. 10.1016/j.techsoc.2025.102917

[B9] Hong L. Ren Q. Liang S. (2016). Comparative study of information service quality of domestic e-commerce website recommendation systems: a case study of Taobao, JD, and Amazon. Libr. Inf. Serv. 60, 97–110. 10.13266/j.issn.0252-3116.2016.23.013

[B10] Huang J. Tong Z. Feng Z. (2022). Geographical POI recommendation for Internet of Things: a federated learning approach using matrix factorization. Int. J. Commun. Syst. e5161. 10.1002/dac.5161

[B11] Hughes A. M. (1994). Strategic Database Marketing. Chicago: Probus Publishing Company.

[B12] Ma L. Sinha N. Cho J. H. D. Kumar S. Achan K. (2023). Personalized diversification of complementary recommendations with user preference in online grocery. Front. Big Data 6:974072. 10.3389/fdata.2023.974072 37034434 PMC10073535

[B13] Müllner P. Lex E. Schedl M. Kowald D. (2023). Differential privacy in collaborative filtering recommender systems: a review. Front. Big Data 6:1249997. 10.3389/fdata.2023.1249997 37901117 PMC10601453

[B14] Niu D. Hao Y. (2019). Research on personalized recommendation models and applications for retail enterprises based on the RFMQ model. Mod. Bus. 20–22. 10.14097/j.cnki.5392/2019.24.008

[B15] Raza S. Ding C. (2022). News recommender system: a review of recent progress, challenges, and opportunities. Artif. Intell. Rev. 55, 749–800. 10.1007/s10462-021-10043-x 34305252 PMC8294232

[B16] Shinde S. K. Kulkarni U. V. (2011). Hybrid personalized recommender system using fast k-medoids clustering algorithm. J. Adv. Inf. Technol. 2, 152–158. 10.4304/jait.2.3.152-158

[B17] Terui Y. Inoue Y. Hamakawa Y. Tatsumura K. Kudo K.. (2025). Collaborative filtering based on nonnegative/binary matrix factorization. Front. Big Data 7:1599704. 10.3389/fdata.2025.1599704 40799979 PMC12339527

[B18] Wang L. Yang M. (2015). Research on the upgrading of China's sports equipment industry from the perspective of the global value chain. J. Shanghai Univ. Sport 39, 5–10. 10.16099/j.cnki.jsus.2015.02.002

[B19] Wu J. Shi L. Yang L. Niu X. Li Y. Cui X.. (2021). User value identification based on improved RFM model and K-means++ algorithm for complex data analysis. Wirel. Commun. Mobile Comput. 2021:9982484. 10.1155/2021/9982484

[B20] Zaghloul M. Barakat S. Rezk A. (2025). Enhancing customer retention in Online Retail through churn prediction: a hybrid RFM, K-means, and deep neural network approach. Expert Syst. Appl. 290:128465. 10.1016/j.eswa.2025.128465

[B21] Zhou Q. Yang W. (2020). Research hotspots and implications of user models in recommendation systems: a knowledge graph analysis based on core literature from the past decade. Inf. Sci. 38, 166–173. 10.13833/j.issn.1007-7634.2020.09.025

[B22] Zong Y. Xing H. (2021). Customer stratification theory and value evaluation—analysis based on improved RFM model. J. Intell. Fuzzy Syst. 40, 4155–4167. 10.3233/JIFS-200737

